# Draft Genome Sequences of Seven *Chryseobacterium* Type Strains

**DOI:** 10.1128/MRA.01518-18

**Published:** 2019-01-03

**Authors:** Andisiwe Matu, Adeline Lum Nde, Lize Oosthuizen, Arina Hitzeroth, Moira Badenhorst, Lebogang Duba, Mphumzi Gidaga, Johan Klinck, Ina-Maré Kriek, Palesa June Lekoma, Lynette Nel, Sérgio Mendes dos Ramos, Jacques Rossouw, Nicolaas Salomane, Neo Segone, Shiela Serobe, Tarsisius Tiyani, Celia J. Hugo, Jeffrey D. Newman

**Affiliations:** a Department of Microbial, Biochemical and Food Biotechnology, University of the Free State, Bloemfontein, Free State, South Africa; b Biology Department, Lycoming College, Williamsport, Pennsylvania, USA; University of Arizona

## Abstract

In an honors course on “Omics Sciences,” draft genome sequences of Chryseobacterium elymi KCTC 22547^T^, Chryseobacterium flavum KCTC 12877^T^, Chryseobacterium hispanicum KCTC 22104^T^, Chryseobacterium lathyri KCTC 22544^T^, “*Candidatus* Chryseobacterium massiliae” CCUG 51329^T^, Chryseobacterium piscium CCUG 51923^T^, and Chryseobacterium rhizosphaerae KCTC 22548^T^ were generated to facilitate phylogenomic comparisons within the genus.

## ANNOUNCEMENT

*Chryseobacterium* species are chemoorganotrophic, Gram-negative rods with flexirubin pigments that give the colonies a yellow-orange color. The genus contains more than 100 described species from diverse habitats, including freshwater sources, soil, marine fish, and human hosts (1–6). Our laboratories have isolated many chryseobacteria, including novel strains, from freshwater creeks and from poultry.

To determine whether a bacterium is a member if an existing species or a novel taxon, it should be compared to previously described species at the genomic level in which an average nucleotide identity (ANI) below 95% signifies that they are different species (7). Genome sequences are now required to describe new taxa, and the Genomic Encyclopedia of Bacteria and Archaea (GEBA) project is sequencing type strain genomes for previously described species (8–11). Indeed, many *Chryseobacterium* type strain genomes have been sequenced as part of GEBA and also as part of the Genome Consortium for Active Teaching Next Generation Sequencing group (GCAT-SEEK) (12, 13). To provide a course-based undergraduate research experience (CURE) (14, 15) for the bioinformatics and -omics sciences honors course (BOCB 6834) at the University of the Free State (UFS) in Bloemfontein, South Africa, we selected seven unsequenced *Chryseobacterium* type strains from the UFS bacteria collection for analysis. Freeze-dried strains in vials were rehydrated with tryptic soy broth and streaked onto tryptic soy agar plates. After 2 days of incubation at 30°C, approximately 10 mg wet cell mass was scraped from the plates and DNA was extracted using the Zymo Quick-DNA fungal/bacterial miniprep kit according to the manufacturer’s recommendations. The purified DNA was assessed for quality by gel electrophoresis and the amount of DNA was quantified by fluorescent dye binding using a Qubit fluorometer (ThermoFisher). The source organisms’ identities were confirmed by amplification of the 16S rRNA genes from the purified DNA with universal primers 27f and 1492r (6) followed by Sanger sequencing of the amplicons with primer 27f and comparison to the type strain database on EzBioCloud (16). Nextera XT Libraries (Illumina) were prepared and 300-base paired-end sequences were generated on an Illumina MiSeq instrument at the UFS Next Generation Sequencing Unit. Reads were assembled using SPAdes v 3.10.0 with default parameters as implemented on PATRIC (17), and the assembly was assessed for quality with CheckM v 1.0.8 using the default parameters (18) on the KBase platform (19) and annotated by the NCBI Prokaryotic Genome Annotation Pipeline (PGAP) (20). The genomes ranged in size from 3.78 to 4.86 Mbp and had G+C mol% values from 33.6 to 37.1% which were all within 0.7% of values measured by HPLC or the thermal denaturation method (Table 1). Comparison of the genome-derived 16S rRNA sequences to the original sequences in GenBank revealed that while most of the originally deposited sequences were fairly accurate, the Chryseobacterium flavum CW-E 2^T^ sequence with the GenBank accession number EF154516 had 25 differences in a small region from position 974 to 1139 relative to the genome-derived sequence and was also very different from all other *Chryseobacterium* 16S rRNA sequences. The authors of the C. flavum species description (3) also noted that the 16S rRNA sequence similarity to the closest relatives was below 95%. The C. flavum KCTC 12877^T^ genome-derived 16S rRNA sequence was most similar (99.1%) to that of Chryseobacterium gleum ATCC 35910^T^, so the ANI was calculated using the Ortho-ANI Tool (21) and found to be 83.2%, confirming that C. flavum and C. gleum are indeed distinct species. The C. flavum KCTC 12877^T^ 16S rRNA sequence was deposited separately into GenBank under the accession number MK116543.

**TABLE 1:** Genome sequence information

Organism (reference)	WGS accession no.	Genome size (bp)	G+C observed/ predicted (mol%)	Coverage (×)	No. of contigs	*N*_50_ (kbp)	No. of 16S rRNA differences^*a*^	CheckM completeness/ contamination
Chryseobacterium elymi KCTC 22547^T^ (1)	QNUH01	4,591,259	37.1/36.4	34	80	249	0	100/0.245
Chryseobacterium flavum KCTC 12877^T^ (3)	QNUE01	4,470,829	36.7/37.2	29	40	311	25	100/0.49
Chryseobacterium hispanicum KCTC 22104^T^ (4)	QNUG01	3,777,625	34.6/34.3	36	148	90	4	100/0.0
Chryseobacterium lathyri KCTC 22544^T^ (1)	QNFY01	4,857,652	37.0/36.6	28	56	305	1	100/0.245
“*Candidatus* Chryseobacterium massiliae” CCUG 51329^T^ (5)	QNVU01	4,409,139	36.0/ND^*b*^	29	116	79	0	100/0.49
Chryseobacterium piscium CCUG 51923^T^ (2)	QNVS01	4,319,594	33.6/33.6	30	158	51	0	100/0.0
Chryseobacterium rhizosphaerae KCTC 22548^T^ (1)	QNUF01	5,267,751	36.3/35.9	22	98	148	1	100/0.245

a Differences between genome-derived 16S rRNA sequences and corresponding GenBank database entries.

b ND, not determined.

### Data availability.

The *Chryseobacterium* whole-genome shotgun (WGS) projects were deposited under the GenBank accession numbers QNUH00000000 (*C. elymi* KCTC 22547^T^), QNUE00000000 (*C. flavum* KCTC 12877^T^), QNUG00000000 (*C. hispanicum* KCTC 22104^T^), QNFY00000000 (*C. lathyri* KCTC 22544^T^), QNVU00000000 (“*Ca.* C. massiliae” CCUG 51329^T^), QNVS00000000 (*C. piscium* CCUG 51923^T^), and QNUF00000000 (*C. rhizosphaerae* KCTC 22544^T^). The versions described in this paper are the first versions, QNUH01000000, QNUE01000000, QNUG01000000, QNFY01000000, QNVU01000000, QNVS01000000, and QNUF01000000, respectively. The Sequence Read Archive (SRA) accession numbers are SRX5025940, SRX5025949, SRX5025952, SRX5026294, SRX5026665, SRX5027813, and SRX5027959, respectively.
